# Correction of 4^th^ and 5^th^ metacarpal synostosis in a skeletally mature hand using de-rotational osteotomies

**DOI:** 10.1080/23320885.2021.2011290

**Published:** 2021-12-16

**Authors:** Christopher D. Liao, Feras Yamin, Roger L. Simpson

**Affiliations:** Division of Plastic and Reconstructive Surgery, Nassau University Medical Center, East Meadow, NY, USA

**Keywords:** Congenital hand deformity, metacarpal synostosis, rotational osteotomies

## Abstract

We present the successful surgical treatment and management of metacarpal synostosis in a near-skeletally mature 15-year-old patient, the significance of which is underscored by an updated review of the literature. We additionally outline a reliable surgical approach for patients with similar clinical presentations and disease severity.

## Introduction

### Embryology and genetics

Metacarpal synostosis describes the abnormal union between two adjacent metacarpals during development. This anomaly has been most frequently described as involving the ring and small fingers (i.e. Metacarpal 4-5 fusion, MF4), manifesting as ulnar deviation of the fifth finger, clinodactyly, reduced range of motion (ROM), and metacarpal hypoplasia [1]. Compared to carpal coalitions, isolated metacarpal synostosis is thought to arise from a sporadic inheritance pattern [1]. Notably, studies involving patients with traditional Kallmann syndrome (KS) and FGFR1-dependent KS demonstrate supportive evidence that MF4 arises from an impaired FGF16-FGFR1 interaction [2].

### Epidemiology

The epidemiology of metacarpal synostosis has not been well described, likely due to the condition’s very rare incidence. While the incidence was reported to range from 0.02% to 0.07% [3], the current body of literature is limited to reviews, case reports, and case series. Furthermore, metacarpal synostosis has been described under different names, including ‘absent fifth metacarpal’, ‘congenital fusion’, ‘bilateral ulnar thumbs’, and ‘congenital metacarpal malformation’ [4].

### Classification

Several classification systems have been described for metacarpal synostosis. In 1993, Buck-Gramcko and Wood described a classification scheme relying on the length of synostosis, divided into three subsets [4]. Offering more detailed characterization, Foucher et al. developed an alphabet-based (I, U, Y, k) classification scheme in 2001 to reflect the shape of the synostosis, direction of epiphysis growth, distal finger deformity, webbing, and metacarpal hypoplasia [5]. Most recently, Liu et al. devised a treatment-oriented system to classify isolated 4^th^ and 5^th^ metacarpal synostosis [6]. This scheme divides patients into three groups based on two key pathological features: the 4–5^th^ intermetacarpal angle and the presence of severe fifth-ray shortening.

In this report, we present the successful surgical treatment and management of a near-skeletally mature 15-year-old patient, resulting in a reliable and stable outcome with low probability of recurrence. For a review of the literature, we conducted a comprehensive search of the PubMed^®^ online database for all publications regarding metacarpal synostosis. Search terms included alternative names such as ‘bilateral ulnar thumbs’, ‘syndactyly type V’, and ‘absent fifth metacarpal’. No retrievable papers were excluded from our literature review; however, only studies that described surgical techniques for correcting 4^th^ and 5^th^ metacarpal synostosis were selected.

## Case REPORT

A 15-year-old right-hand dominant male presented with bilateral deformities of his ring and small fingers that were noted at birth. The patient grew concerned about the appearance and functionality of his hands, stating that his small finger constantly caught on his clothing. He also reported difficulty making a complete fist due to overriding fingers, resulting in decreased grip strength.

Examination of both hands revealed an obvious ulnar deviation of bilateral ring and small fingers with the small fingers held in an exaggerated abducted position and a widened interdigital web space. The ring and small fingers were malrotated ulnarly and radially, respectively, resulting in overriding of the two digits during finger flexion, grip weakness, and incomplete fist formation. The metacarpophalangeal joints (MCPJ) demonstrated normal active and passive ROM. However, radial deviation and tethering of the flexor tendons of the small fingers was appreciated (Figure 1, Video 1). The remaining hand examination was unremarkable.

**Figure 1.**
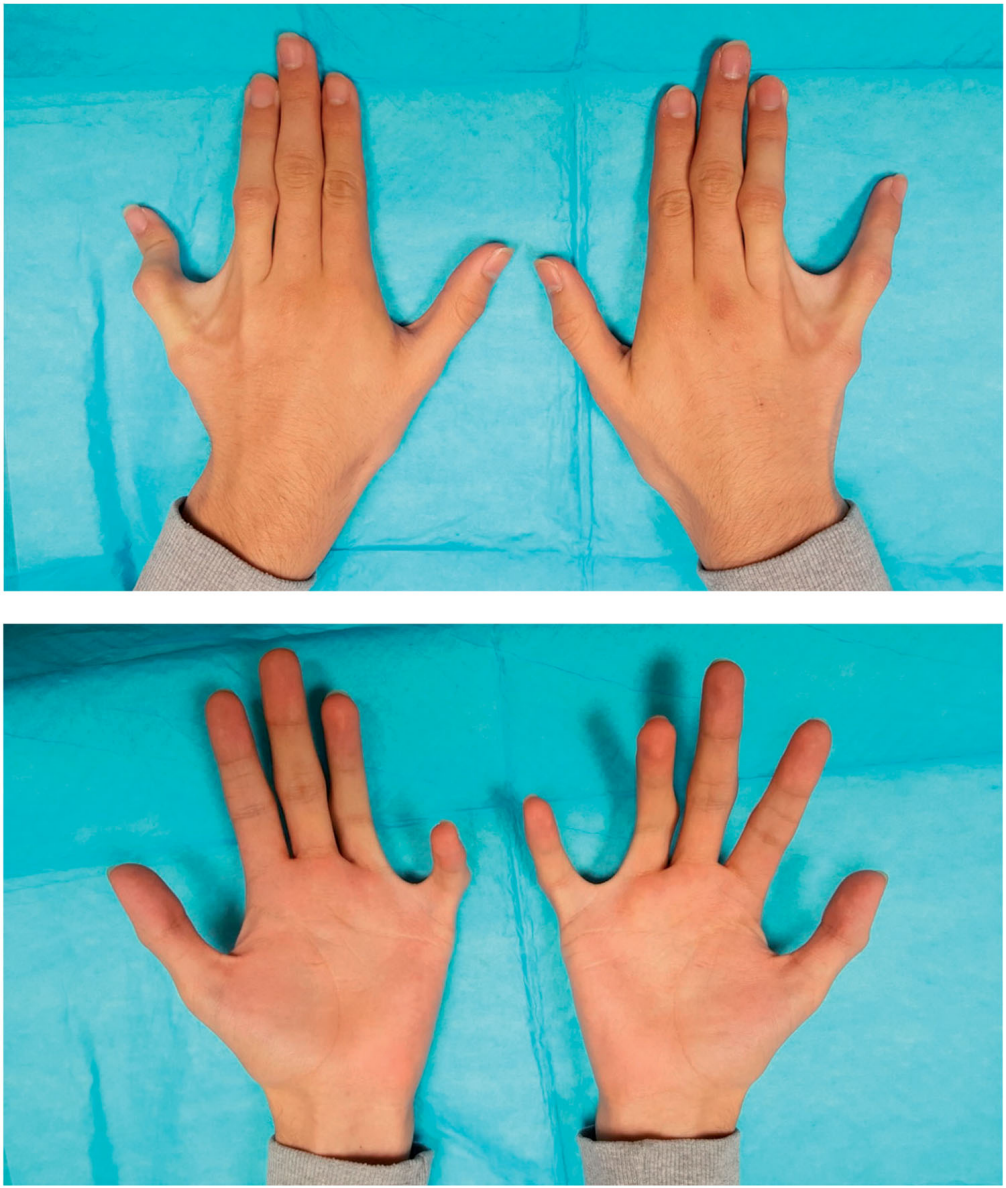
Preoperative photographs of the patient’s left and right hands.

Hand radiographs demonstrated a common metacarpal shared between 4^th^ and 5^th^ digits proximally with asymmetric branches distally, despite having independent MCPJs and osteoepiphyseal surfaces (Figure 2).

**Figure 2.**
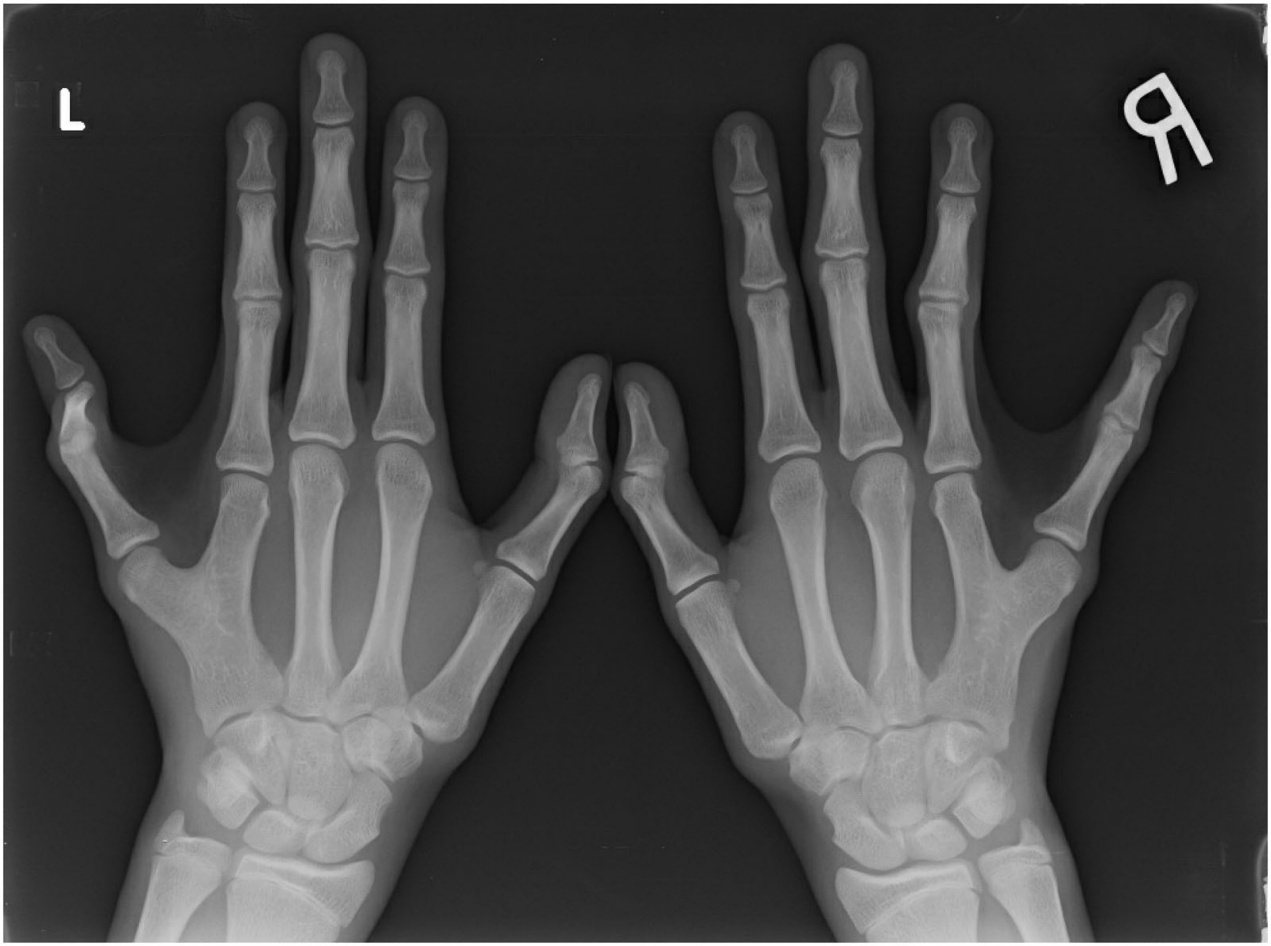
Preoperative radiographs of the patient’s left and right hands.

### Surgical planning and operative details

Preoperatively, we combined several radiographic images to estimate the appropriate degree of angulation and rotation with precise virtual measurements. The procedure was performed under general anesthesia and fluoroscopic guidance. Total operative time was 2 h, during which a right arm tourniquet was applied. A 6-cm incision was created on the dorsal aspect of the ulnar side of the right hand. A surgical plane was dissected between the heads of the 4^th^ and 5^th^ metacarpals. Wedge osteotomies were created using an oscillating saw. A 2-mm closing wedge osteotomy was made on the ulnar side of the neck of the 4^th^ metacarpal at the exact location and orientation dictated by the preoperative diagrams. The head of the 4^th^ metacarpal and ring finger were then de-rotated and realigned in the appropriate position. This was followed by fixation of the osteotomy site with a 1.6-mm mini-plate and 8-mm non-locking screws spanning the osteotomy. Another 2-mm closing wedge osteotomy was created on the radial aspect of the neck of the 5^th^ metacarpal, again adhering to the preoperative diagrams. The small finger was de-rotated and realigned into a more acceptable position anatomically and functionally. The normal cascade of the right hand was restored intraoperatively. The wedge osteotomy bone fragments were placed as grafts to lengthen the 5^th^ metacarpal head, followed by fixation with a 1.6-mm mini-plate and 8-mm non-locking screws to stabilize the construct.

The extensor tendons and digital neurovascular bundles of both digits were returned to their respective anatomic positions. A hemostatic field was achieved using electrocautery. The periosteum was closed with polydioxanone sutures covering the plates and osteotomy sites. The skin was closed with interrupted vertical mattress 4-0 nylon sutures. A bulky dressing and volar splint were placed on the right hand maintaining an intrinsic plus position.

### Postoperative Follow-Up

Postoperatively, the patient progressed appropriately. Two weeks after surgery, the nylon sutures were removed, revealing a completely healed surgical incision. The following week, full active ROM therapy was initiated. On examination, the right small and ring fingers exhibited improved alignment with no evidence of malrotation during flexion (Figure 3(A,B), Video 2).

**Figure 3.**
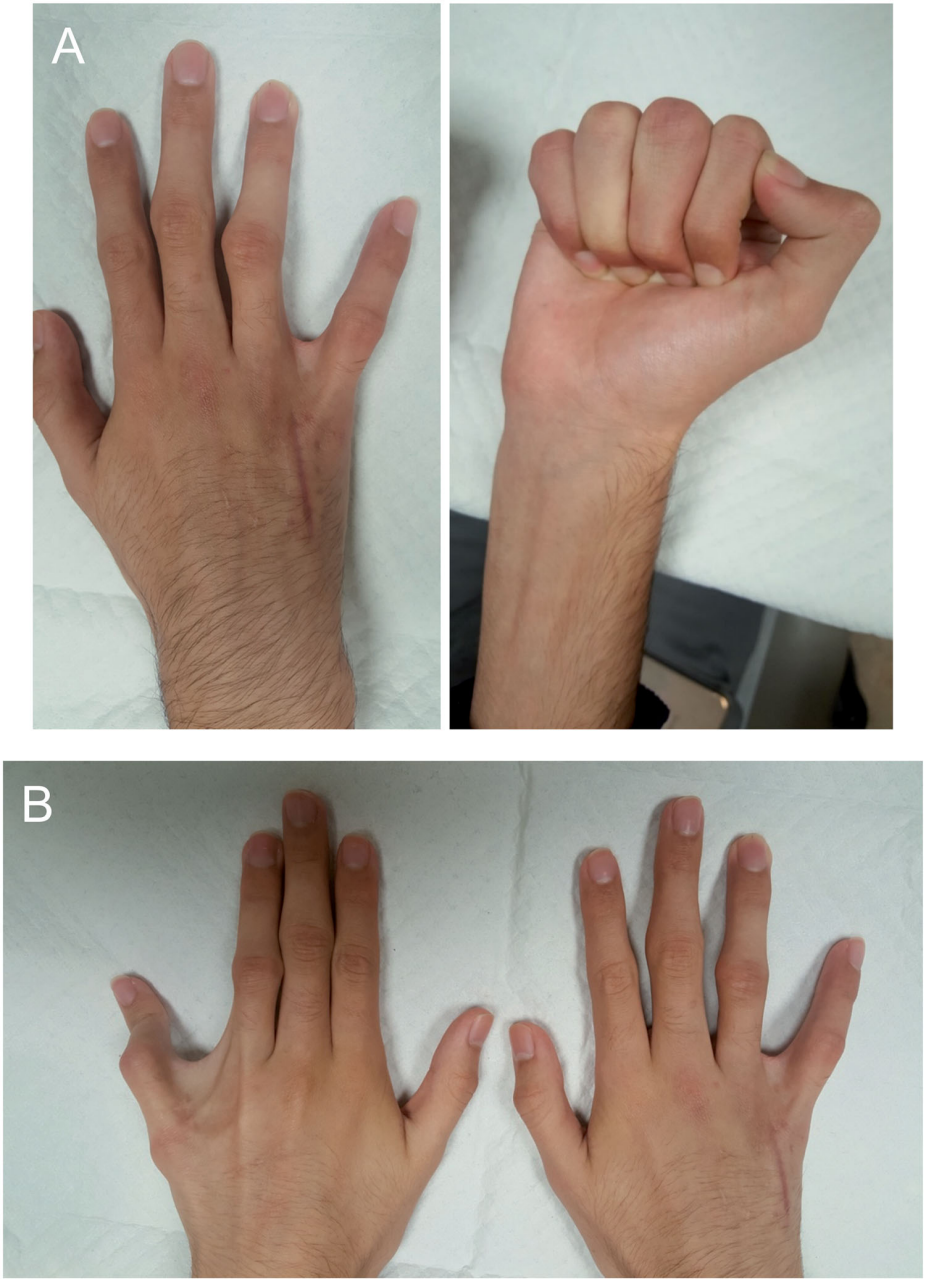
Postoperative photographs of the patient’s (A) right hand and (B) both hands taken at 4 months after surgery. The left hand has not been surgically corrected.

Radiographic images captured 3 months after surgery confirmed well-healed right 4^th^ and 5^th^ metacarpals at osteotomy sites with stable fixation plates and well-incorporated bone grafts (Figure 4). During this visit, the patient regained full ROM and strength of his right hand. The orientation of his right small and ring fingers appeared functionally and aesthetically acceptable. The patient was satisfied with his ability to form a complete fist and a powerful grip, while denying previous concerns that he reported prior to surgery.

**Figure 4.**
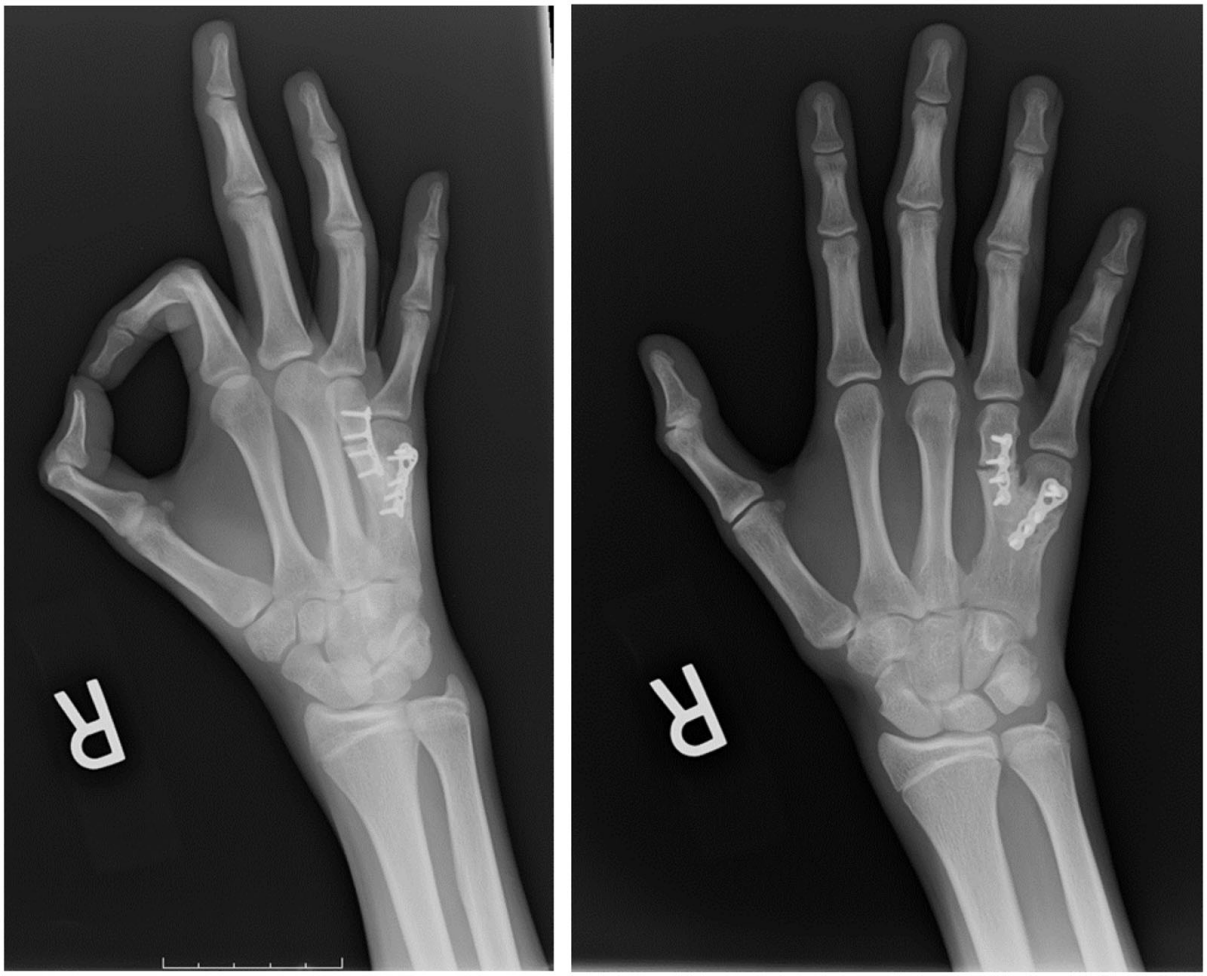
Postoperative radiographs of the patient’s right hand taken at 4 months after surgery.

## Discussion

Our literature review identified six case series and five case reports documenting 4^th^ and 5^th^ finger metacarpal synostosis. The total number of patients was 178, and the ages ranged from 1 month to 20 years (Table 1). One unique element of the current case presentation is the age of the patient and his proximity to full skeletal maturity. There is limited data in the literature regarding surgical treatment of metacarpal synostosis in skeletally mature patients; our review identified only three documented cases of patients older than age 15 [7,8].

**Table 1.** Summary of literature regarding 4th and 5th metacarpal synostosis.

Reference	Number of patients (Hands)	Sex (M:F)	Age at operation (Range)	Follow-up (Range)	Description of surgical techniques
Buck-Gramcko and Wood [4]	109 (152)	61:48	n/s	n/s	Osteotomy with bone block graft; supplementation with tendon transposition, insertion of silicone rubber sheeting, removal of accessory digits, and collateral ligament reconstruction
Foucher et al. 2001 [5]	20	22:6	n/s	Average: 3 yrs (1 to 6 yrs)	- Type Ys: Reverse trapezoidal bone graft - Type Ya and k: Longitudinal osteotomy and interposition (inadequate results acknowledged) - Type I: separation is risky for MCPJ mobility
Gottschalk et al. 2012 [10]	6 (8)	2.3:1	Median: 5 yrs (2 to 16 yrs)	Average: 3 yrs (1 to 14 yrs)	Longitudinal osteotomy and bone graft substitute interposition (coralline hydroxyapatite)
Horii et al. 1998 [11]	12 (15)	n/s	4.4 yrs	Average: 10.3 yrs (1 to 20 yrs)	Osteotomy with bone graft using silicone block
Liu et al. [6]	12 (16)	10:2	Median: 6 yrs (3 to 13 yrs)	n/s	- No treatment for Type A narrow IMA - Opening wedge osteotomy, triangular or trapezoidal bone graft harvested from synostosis site
Miura [7]	14 (17)	11:14	Average: 4 yrs (1 month to 20 yrs)	n/s	Osteotomy with intermetacarpal block graft (silicone block or iliac bone graft)
Hooper and Lamb 1983 [12]	1 (2)	n/s	18 mos	18 mos	Oblique osteotomy with K-wire fixation
Jianmongkol et al. [8]	1 (1)	M	10 yrs	5 yrs	Two osteotomies (oblique-transverse + vertical) with two bone blocks placed between osteotomy sites followed by K-wire fixation
Kawabata et al. [9]	1 (2)	M	12 yrs	1.5 yrs	Partial osteotomy of 5th metacarpal with application of mini-Hoffmann lengthener and interpositional washer followed by K-wire fixation; hemicallotasis at 0.5 mm/day
Yamamoto et al. 2000 [13]	1 (1)	F	12 yrs	1 year	Wedge osteotomy from bifurcate portion of fused metacarpal base with insertion of harvested bone block followed by K-wire fixation
Yildirim et al. 2003 [14]	1 (1)	F	10 yrs	n/s	Transverse osteotomy and closing wedge osteotomy (greenstick fracture) followed by K-wire fixation

Compared to younger, developing patients, our patient’s presentation enabled more accurate evaluation of the functional and anatomical limitations that this deformity imposes on daily activities. Additionally, we were able to clearly assess the immediate improvement in the patient’s hand function. Given that his metacarpal bones were close to full maturity at the time of surgery, recurrence of synostosis secondary to metacarpal bone growth is unlikely. Therefore, our results underscore the potential clinical benefit of achieving more reliable and stable outcomes when surgical treatment is offered at an older age.

Because of the variability in metacarpal synostoses, numerous treatment strategies have been proposed [3]. Our review demonstrated that the most frequently performed surgical approach involves dividing the bony synostosis and separating the metacarpals with an interpositional spacer (Table 2; Figure 5). Options for the spacer include iliac crest bone graft, silicone rubber, costal cartilage, and bone substitutes [3]. Less commonly, Kirschner-wire (K-wire) fixation, tendon transposition, and Hoffman lengtheners were utilized (Table 2; Figure 5). One report in 2005 described the use of two osteotomies: one oblique-transverse and another vertical osteotomy with the placement of two bone blocks [8]. In 1997, Kawabata et al. proposed a hemicallotasis of the radial cortex of the 5^th^ metacarpal as an alternative lengthening strategy [9].

**Figure 5.**
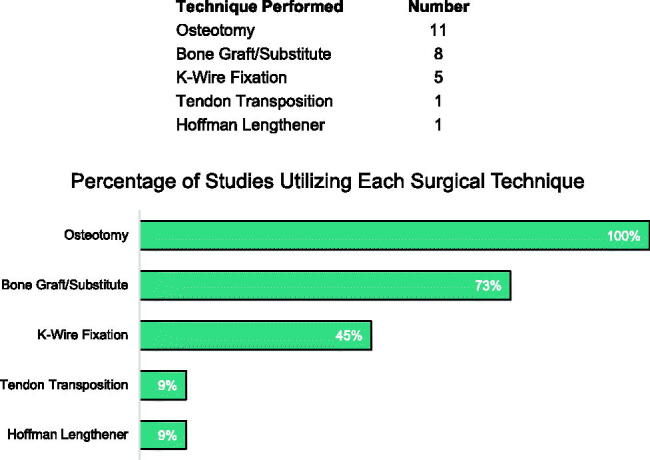
Percentage of studies utilizing each surgical technique. Only one study described the use of bone graft harvested directly from the synostosis site.

**Table 2.** Summary of surgical techniques to treat metacarpal synostosis.

Reference	Osteotomy	Bone graft substitute or spacer	Bone graft harvested from synostosis site	K-Wire fixation	Tendon Transposition	Hoffman Lengthener
Buck-Gramcko and Wood [4]	x	x			x	
Foucher et al. 2001 [5]	x	x				
Gottschalk et al. 2012 [10]	x	x				
Horii et al. 1998 [11]	x	x				
Liu et al. [6]	x		x			
Miura [7]	x	x				
Hooper and Lamb 1983 [12]	x			x		
Jianmongkol et al. [8]	x	x		x		
Kawabata et al. [9]	x			x		x
Yamamoto et al. 2000 [13]	x	x		x		
Yildirim et al. 2003 [14]	x			x		

There are currently no concrete guidelines for treatment of metacarpal synostosis, which is complicated by the absence of a universal, treatment-directed classification scheme. With respect to the three aforementioned classification schemes, the patient described in our report could be best classified as either a Foucher Class-Ya, Buck-Gramcko Wood Class-IIIB, or Type-B1 according to the system devised by Liu et al. [4–6] Within our review, only one study specified the surgical management of Foucher-Ya synostosis using a trapezoidal bone graft and progressive lengthening but admitted a suboptimal cosmetic outcome [5]. In the original publication by Buck-Gramcko and Wood, there was no specific surgical approach outlined for Class-IIIB synostoses [4]. For Type-B1 synostoses, Liu et al. reported satisfactory results after utilizing an opening wedge adduction osteotomy of the 5^th^ metacarpal with bone grafting and an additional wedge osteotomy of the 4^th^ metacarpal [6].

In comparison, our surgical technique involved wedge and de-rotational osteotomies combined with bone grafting and plate fixation, the latter of which adds another unique element to our case. We anticipate that the plate will reinforce and provide long-term stability for the reconstruction. Overall, we outline a reliable surgical approach after classifying a case of 4^th^ and 5^th^ finger metacarpal synostosis according to three published classification schemes. Given the variability in presentation and outcomes of metacarpal synostosis, future studies adequately characterizing disease type and severity will be necessary to develop improved surgical strategies.

## Supplementary Material

Supplemental Material
