# Supplementary material for: Correction of 4th and 5th metacarpal synostosis in a skeletally mature hand using de-rotational osteotomies
Source: Case Reports Plast Surg Hand Surg. 2021 Dec 16;9(1):15–21. doi: 10.1080/23320885.2021.2011290 (PMC8725938; doi:10.1080/23320885.2021.2011290)

**VIDEOS**

Video 1 (still). Preoperative video of hands.


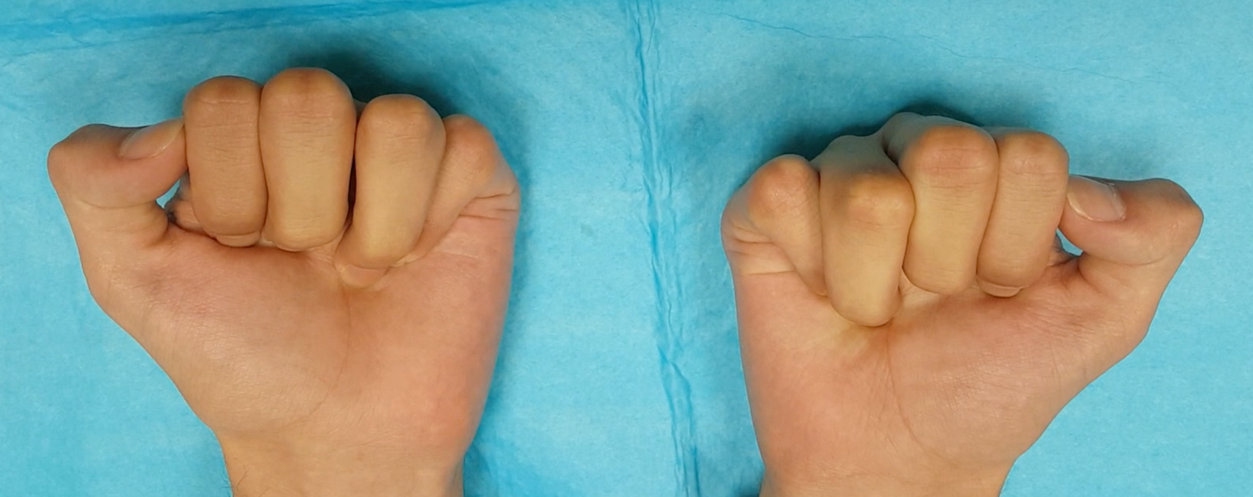


Video 2 (still). Postoperative video of hands. The left hand has not been surgically corrected.


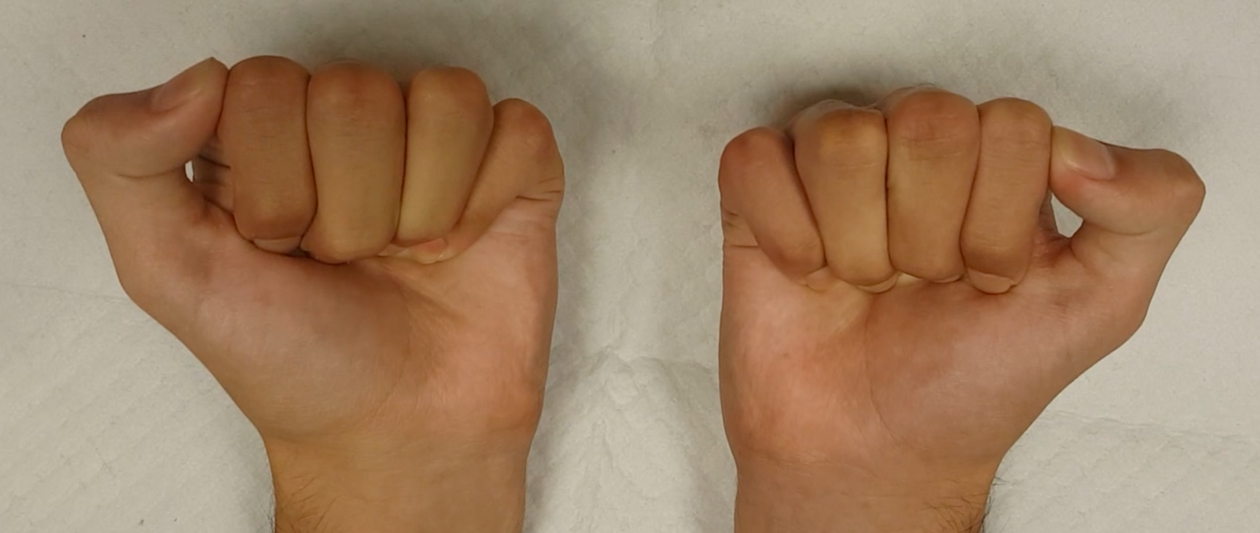

Supplement: Supplemental Material [file ICRP_A_2011290_SM5333.zip › suppl_data/Video Stills 1 and 2.docx]
